# The ACHIEVE Program: Bringing Chicago Youth and Community Organizations Together to Impact Local Disparities

**DOI:** 10.1007/s10900-024-01357-2

**Published:** 2024-04-14

**Authors:** Monica Kowalczyk, Jeronimo Najarro, LaTonya Hill, Todd Barnett, Anna Volerman

**Affiliations:** 1 University of Chicago Biological Sciences Division, Chicago, IL USA; 2 University of Chicago Charter School, Chicago, IL USA

**Keywords:** Community-based, Community engagement, Disparities, Youth

## Abstract

To evaluate the Advancing Community Health and Individual leadership through a noVel Educational (ACHIEVE) program uniting Chicago high school and undergraduate students (scholars) and community organizations to empower youth to meaningfully impact communities while enhancing organizational capacity. Between 2020 and 2022, the ACHIEVE program engaged cohorts of youth in classroom-based learning and community-based projects targeting health and education disparities. Pre and post-program surveys were administered to scholars to assess knowledge about disparities, skills, and self-efficacy. Semi-structured interviews were conducted with community organization leaders to examine programmatic impact. Descriptive and thematic analyses were performed. Across four cohorts (March 2020; September 2020-May 2021; September-November 2021; March-May 2022), 85 students participated in the ACHIEVE program. Scholars supported 19 community-based projects that increased awareness of local issues and resources and evaluated programs. Scholars reported advancement in their knowledge and skills as well as interest in sustaining their community engagement. Leaders shared several benefits at the organizational and community levels from collaborating with scholars. The ACHIEVE program enabled bidirectional learning between scholars and organizations. It also demonstrated that youth can contribute positively to addressing disparities while supporting local organizations and communities.

## Introduction

Disparities in health and education are prominent in Chicago. Studies show a significant gap in life expectancy between Chicago neighborhoods, including as much as a 30-year difference between Englewood in the South Side and Streeterville near the Chicago Loop [1]. Marginalized neighborhoods, like Englewood, have predominantly Black or Hispanic/Latino populations and limited opportunities and resources [2] on top of longstanding structural factors, resulting in poorer educational [3] and health outcomes for youth and adults [4]. Within these most vulnerable neighborhoods, community involvement is critical for tailored and comprehensive solutions to advance equity. Such engagement fosters empowerment and development of knowledge, skills, and confidence among community members to engage in regeneration activities [5]. 

Young people, including adolescents and young adults, can play an important role in developing and implementing sustainable and effective community programming. Youth, especially those of color, are often neglected from such efforts, however many consider it highly important to give back to their communities [6]. They bring valuable insights from their lived experiences and can be more influential within their community compared with professionals outside of the community [7]. In addition, engaging young people can inspire them to become agents of change in their communities and contribute to their personal growth. Such efforts can apply the Positive Youth Development framework that intentionally engages youth, leverages their strengths and, ultimately, promotes positive outcomes among youth [8]. Outcomes for youth engagement in communities can include improved social, emotional, and cognitive competencies, increased self-efficacy, and enhanced contribution to communities among young people [8, 9]. Youth programs that incorporate skill development and offer hands-on experiences in community initiatives can positively impact youth and contribute to efforts to reduce local disparities [10]. 

Despite efforts focused on community development in Chicago’s vulnerable neighborhoods, gaps persist in such initiatives. For instance, local organizations that aim to address the needs of Chicago’s high-burden communities have limited capacity [11, 12]. Additionally, Chicago youth lack engagement and empowerment to meaningfully contribute to their communities and the workforce. Data shows that 13.4% of young people (16–24 years old) in Chicago were not engaged in school and work, with Black youth disproportionately impacted (25.8%) [13].

In response to these unmet needs, the **A**dvancing **C**ommunity **H**ealth and **I**ndividual L**E**adership Through a No**V**el **E**ducational (ACHIEVE) program was established to foster collaborations between Chicago youth and community organizations to address local issues. The program equipped participating youth, called ACHIEVE scholars, with education about local issues, knowledge and skills applicable to projects, as well as exposure to workforce opportunities, empowering them to become agents of change within their communities. ACHIEVE scholars collaborated with community organizations to boost the workforce capacity to complete mission-driven projects focused on local issues. These projects leveraged the assets of the scholars while honoring and incorporating their lived experiences and interests to support the work of partnering organizations. This article describes the ACHIEVE program and presents results of a mixed-methods analysis that evaluated the program’s impact on both scholars and community partners. The outcomes of the program may inspire other communities to involve and empower youth in community development.

## Methods

### Program Overview

The ACHIEVE program uniquely brought together high school students from marginalized communities on Chicago’s South Side and undergraduate students from the University of Chicago in civic engagement and leadership with a focus on health and education disparities. The program objectives were to: (1) educate high school and undergraduate students about topics at the intersection of education and health; (2) cultivate leadership and non-cognitive skills among students to prepare them for the workforce; and (3) engage students in civic engagement within communities.

The program was developed in 2019 as part of a collaboration between the University of Chicago Medicine and the University of Chicago Charter School in response to identified community and school needs [14]. The ACHIEVE program leaders were a physician faculty member at the University of Chicago Medicine, who designed the curriculum and led program implementation, and a senior director of community engagement and partnerships at the University of Chicago Charter Schools, who formed and sustained partnerships with local high schools and organizations in Chicago’s South Side. An advisory committee comprised of community, school, health system, and university stakeholders guided program development to help ensure the ACHIEVE program was relevant to the communities served.

The ACHIEVE program recruited high school and undergraduate students using a multi-pronged approach. Flyers were developed with information about program goals, components, and dates. For high school students, school counselors and administrators distributed the flyers through targeted efforts in their schools. For undergraduate students, flyers were distributed through health-related programs and student organizations within the University of Chicago. Interested students completed an application with questions about demographic characteristics, extracurricular activities, plans and goals after high school or college graduation, interest in the program, and references. Program leaders reviewed applications and selected candidates for interviews. One-on-one interviews were held with applicants to learn about the candidates as well as answer questions about the program. Key selection criteria included a demonstrated passion for community involvement, interest in health and/or education as a professional career, and ability to make a full commitment to the program. Students selected as ACHIEVE scholars were asked to sign a program agreement to indicate their commitment to the 5–8 h per week to the ACHIEVE program. For high school students under 18 years old, parents/guardians were asked to sign the program agreement as well.

The ACHIEVE program utilized a multi-component curriculum that included classroom-based learning and community-based projects to support its overall goals. For classroom-based learning, scholars participated in seminars and workshops to learn more about local disparities and to develop their leadership and research skills, respectively. Specific topics discussed related to education and health included: disparities, nutrition and food insecurity, physical education, childhood chronic diseases, mental health, substance use, and community violence. The skill development focused on goal setting and planning, project management, teamwork and collaboration, verbal communication skills, professional writing, science communication, and research methods.

To support hands-on experiences for scholars, the ACHIEVE program leaders partnered with local organizations to identify and develop community-based projects that advanced the organization’s mission while also engaging scholars in their communities and cultivating leadership and professional skills (e.g., project management, data collection, data analysis). When developing projects, an information sheet was created with key organization information (e.g., mission, director, contact) and project information (e.g., topic, background, deliverables). Project teams consisted of three to five scholars grouped based on their interests and skills. In these project teams, undergraduate scholars served as mentors to high school scholars. Teams were responsible for carrying out the projects and completing the deliverables, as well as maintaining regular communication and progress updates with the community organizations through emails, calls, and meetings.

**Fig. 1:**
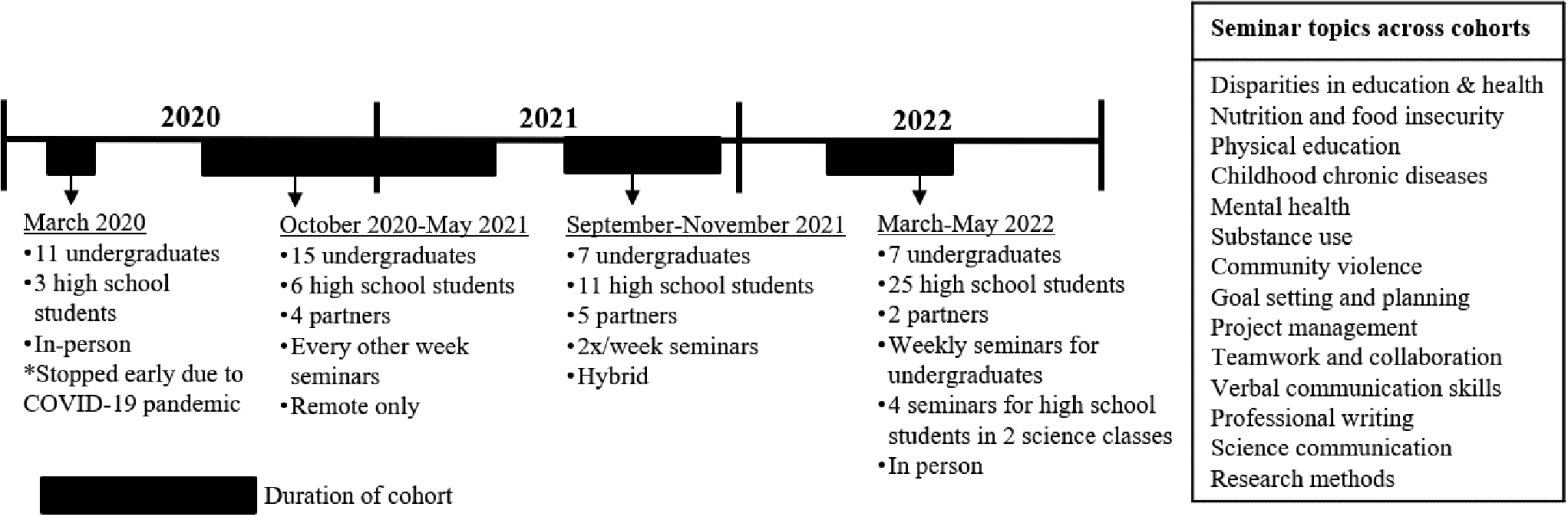
Timeline of the ACHIEVE program (2020-2022)

The ACHIEVE program was held from spring 2020 to spring 2022 with four cohorts during that time: March 2020; September 2020-May 2021; September-November 2021; and March-May 2022 (Fig. 1). Due to the COVID-19 pandemic as well as feedback from ACHIEVE scholars and community partners, program implementation was adapted across its duration, including changes to program length, cohort size, meeting structure (in-person, remote, hybrid), and seminar topics.

In the early cohorts, one challenge encountered in the ACHIEVE program was inconsistent attendance among the high school scholars due to their limited capacity. These limitations to full participation arose from various factors, including responsibilities of higher priority including schoolwork, employment, and family obligations. Consequently, we addressed this challenge by offering high school scholars service-learning hours to enhance student engagement and completion rates. We also attempted to offer financial stipends to improve participation and alleviate financial burdens but were not able to do so for administrative reasons. To address this issue, we integrated the ACHIEVE program into a high school science class in May of 2022 with undergraduate ACHIEVE scholars serving as teaching assistants. While the goals of the program were similar, its format and scope were significantly different; as a result, we did not distribute the same pre- and post-program surveys to this group of high school students.

### Program Evaluation

To assess the program’s impact on scholar outcomes, high school and undergraduate scholars were asked to complete surveys immediately before the start and at the end of their session. The pre-program survey included 24 questions using a five-point Likert scale that assessed various topics: scholars’ knowledge about health and education disparities; skills including leadership, communication, and research; and self-efficacy in impacting local communities (Table 1). The post-program survey included 24 questions, also using a five-point Likert scale, that assessed scholars’ confidence in these same competencies after completing the program (Table 1). Pre- and post-program surveys also included open-response questions that asked scholars to reflect on their experience in the ACHIEVE program, including what they learned in the program, their confidence in executing program competencies, as well as the actions they will take after the program. Surveys were distributed and completed via REDCap (Nashville, TN).

**Table 1:** Competencies of scholars pre- and post-participation in the ACHIEVE program

Knowledge, skills, and self-efficacy to meaningfully impact communities				% scholars reporting agreement pre-program (*n* = 57)			% scholars reporting more post versus pre-program (*n* = 36)	
“I am knowledgeable about health and education issues”				71.9			91.4	
“I can explain how different aspects of my community can influence health”				73.7			91.4	
“I know how to collect information about health issues that affect my community”				26.3			94.3	
“I know how to speak aloud in class about health issues in my community”				50.9			94.3	
“I know how to work with others to improve the health of my community”				29.8			91.4	
“I am interested in advocacy focused on health and education”				100			88.6	
“I feel that I can lead positive change in my community/school”				82.5			77.0	
“I am confident in my abilities to reduce health problems in my community”				52.6			82.9	
“I am confident in my abilities to speak in classes, groups, or public places”				70.2			71.4	
**Skills in research and communication**				% scholars reporting confidence pre-program (*n* = 57)			% scholars reporting more confidence post versus pre-program (*n* = 35)	
Conduct a literature search to find information about a science topic				94.7			80.0	
Interpret data such as graphs and charts				89.5			57.1	
Engage with community leaders about health and education issues				59.6			97.1	
Communicate my research in an oral presentation				80.7			77.1	
**Skills in project management and teamwork**				% scholars reporting confidence pre-program (*n* = 57)			% scholars reporting more confidence post versus pre-program (*n* = 35)	
Working in a team on a project				94.7			85.7	
Developing and articulating a shared vision, roles, and responsibilities in a group				89.5			82.9	
Setting goals and deadlines and meeting them				91.2			80.0	
Seeking out and using resources				84.2			85.7	
Taking initiative to address an issue with a project				80.7			91.0	
Recognizing the strengths of others on a team				89.5			88.6	
Learning from others in a team environment				94.7			91.4	
Providing opinion and feedback when working in a group				86.0			85.7	
Actively listening to others and encouraging contributions from everyone				96.5			85.7	
Being mindful about how personal attitudes, beliefs, and experiences influence work				96.5			85.7	
Valuing and honoring diverse perspectives				96.5			91.4	

To assess the program’s impact on the community organizations and local communities, semi-structured interviews were conducted with the leaders of community organizations who partnered with the ACHIEVE program between 2020 and 2022. An interview guide was utilized with questions about the goals of the partnership, experiences in collaborating with the ACHIEVE scholars on the project, and impact of the deliverables on the organization and community. Interviews were conducted by the program manager via Zoom and recorded. Key points and quotes from the interviews were transcribed and de-identified.

### Data Analysis

A mixed methods analysis was performed to evaluate the impact of the ACHIEVE program on scholars and the community. Descriptive statistics were completed for Likert scale questions on the pre- and post-program surveys, including the mean and the percentage of participants who responded with agreement or confidence (i.e., 4 or 5) on the scale. Thematic analysis was applied for the responses to open-ended questions from the pre- and post-program surveys as well as interview questions [15]. Two authors iteratively read and categorized responses for each question into themes and subthemes. Discrepancies were resolved through discussion.

## Results

Across the four cohorts, a total of 85 scholars participated in the ACHIEVE program, including 45 undergraduate students and 40 high school students (Table 2). The pre-program survey was completed by 57 participants (n *=* 18 high school scholars, *n* = 39 undergraduate scholars). The post-program survey was completed by 36 participants (*n* = 9 high school scholars, *n* = 27 undergraduate scholars). The 25 high school students who participated in the program within their science class did not complete the same surveys given the different scope of that program. Among participants, a majority were female (66.7%, *n* = 40/60). Nearly all high school students were Black, consistent with the South Side community where the high schools were located. The race/ethnicity of the undergraduate students varied.

**Table 2:** Demographics of ACHIEVE program scholars, 2020–2022

	March 2020 (n = 14)	September 2020 - May 2021 (n = 21)	September - November 2021 (n = 18)	March - May 2022 (n = 32)	Total (n = 85)
**Educational level of scholars**					
High school students	3	6	11	25	45
Undergraduate students	11	15	7	7	40
**Genders reported by scholars** ^**a**^					
Female	9	11	15	5	40
Male	4	3	3	2	12
Other	1	0	0	0	1
Not reported	0	7	0	25	32
**Race/ethnicity reported by scholars** ^**b**^					
American Indian/Alaska Native	0	0	0	0	0
Asian	0	0	7	5	12
Black/African American	0	0	10	0	10
Hispanic/Latino	0	0	1	1	2
Native Hawaiian/Other Pacific Islander	0	0	0	0	0
White	0	0	0	1	1
Not reported	14	21	0	25	60

^a^Gender identity data not collected for high school scholars for the September 2020-May 2021 (n = 6) and March-May 2022 (n = 25) cohorts

^b^Race/ethnicity were not asked in the program surveys for the March 2020 and September 2020-May 2021 cohorts. Race/ethnicity was not asked to the high school students in March-May 2022

The ACHIEVE program engaged with community organizations in varied disciplines, including community development, early literacy, lung health, nutrition, and youth development. Through these partnerships, scholars supported 19 community-based projects across 8 organizations between 2022 and 2022. Some projects aimed to increase awareness of community issues, including the digital divide during the pandemic, importance of vaccination, racial practices in lung measurement, and secondhand exposure to marijuana smoke; for these projects, scholars researched the topics and produced issue briefs and other summary documents. Additionally, some projects focused on increasing access to resources, for example related to social emotional learning and community gardens, with scholars producing and distributing infographics with key information on the topic. Lastly, some projects involved programmatic evaluation in which scholars developed surveys and interview guides as well as collected and analyzed data about the programs’ impacts.

### Scholar Outcomes

At the start of the ACHIEVE program, scholars reported varied knowledge, skills, and self-efficacy to meaningfully impact communities (Table 1). Most scholars reported they were knowledgeable about health and education issues (71.9%), able to explain how different aspects of their community can influence health (70.3%), and able to lead positive changes in their school or community (82.5%). For research, communication, and leadership skills, most scholars reported confidence in conducting a literature search (94.7%), interpreting data such as graphs and charts (89.5%), working in a team on a project (94.7%), setting and meeting goals and deadlines (91.2%), as well as speaking in classes, groups, or public places (70.2%). Few scholars knew how to collect information about health issues affecting communities (26.3%) or how to work with others to improve the health of communities (29.8%). More than half of scholars indicated confidence in their ability to engage with community leaders about health and education issues (59.6%) and to reduce health problems in their communities (52.6%).

After completing the ACHIEVE program, scholars reported enhanced knowledge, skills, and self-efficacy (Table 1). Nearly all scholars reported greater knowledge on health and education issues (91.4%). Scholars also stated they were more knowledgeable about collecting information about health issues in communities (94.3%) and working with others to improve the health of communities (91.4%). Despite initially reporting high competency in research, communication, and leadership skills, scholars indicated that the ACHIEVE program further enhanced such skills. For example, scholars indicated increased confidence in conducting literature searches (80%), engaging with community leaders about health and education issues (97.1%), and communicating their research orally (77.1%).

Based on responses to open-ended questions in the post-program survey, three themes emerged about the program’s effects on scholars: improved skills, enhanced awareness of health and education, and increased desire to sustain engagement (Table 3). First, scholars reported more confidence in skills related to research, communication, and leadership after completing the program. One participant wrote, *“I learned various techniques in collecting information and conveying them effectively.”* Additionally, scholars reported greater awareness of topics around health and education, including careers. One student shared, *“I hope to continue learning about different career paths that really heavily impact the intersections of healthcare and education.”* Finally, scholars expressed interest in continued opportunities to serve marginalized communities, address community issues, and partner with community organizations. One scholar shared, *“I am determined to contribute to the improvement of education in our neighboring communities, promote equitable access to healthcare in my future career.”*

**Table 3:** Individual, organizational, and community outcomes of the ACHIEVE program based on perspective of scholars and community organization leaders

Themes	Subthemes	Illustrative quotes
**Scholar perspectives on individual outcomes**		
Improved skills	Leadership skills	*“Because of ACHIEVE, I am more confident in my ability to lead a team.”* *“I am more confident in sparking conversations with peers, advisors, and experts around these [health and education] topics.”*
	Research skills	*“Through the ACHIEVE program, I learned about creating infographics and how to do research about community health.”* *“I am more confident in my ability to analyze data and display it in new way.”*
	Communication skills	*“I advanced my team-working skills and was able to communicate and work responsibly and efficiently.”* *“I learned about how to communicate with others. At first I was very shy but now I feel more confident.”*
Enhanced awareness of health and education	Health and education disparities	*“I learned about social determinants of health, inequities in education, and strategies for capacity-building with existing non-profit organizations.”* *“I hope to continue learning about ongoing health equity projects within University of Chicago and the larger Chicago community to better understand the concurrent health issues and how I can impact the remaining of my study.”*
	Careers in field	*“I learned about the different, underrated subfields within healthcare and education and how ideas in these subfields can impact individuals in the communities at hand.”* *“After completing ACHIEVE, I hope to continue learning about different career paths that really heavily impact the intersections of healthcare and education.”*
Increased desire to sustain engagement	Serve communities	*“Because of ACHIEVE, I am more determined to seek out opportunities to help local communities.”* *“I am more determined to participate in more opportunities in communities and health and working with others.”*
	Partner with community organizations	*“I am more determined to make contributions to local community through organizations.”* *“I am more determined to work with local organizations to implement efficient and effective programs.”*
	Address community issues	*“I am more determined to be aware of what is going on in my community and to learn how to solve the different problems.”* *“I am more determined to be engaged in Hyde Park and surrounding communities, and be an agent of change throughout my career, hoping to address inequalities in health.”*
**Community partner perspectives on organizational and community outcomes**		
Organizational impact of partnership	Incorporated diverse ideas	*“Being a one-person organization, volunteers are my ‘staff’. It was a team that I got to have for a short period of time that became thought partners. The questions they [ACHIEVE students] were asking became an opportunity to sharpen our programming.”* *“We gave them a broad ‘here’s what you want, you can do anything below it.’ They were really good about asking very specific questions to tailor what they would be giving us. Really it was the questions that they asked that made the end product so great.”*
	Lessened workload for organization	*“If we hadn’t partnered with your team [ACHIEVE scholars], I don’t think we would have gotten this done in such a timely manner.”* *“I love ACHIEVE because to me it’s doing heavy lifting on things that would be really difficult for me to do on my own. The collaborative space in that realm is super helpful because I have a team.”*
	Completed specific deliverables with high quality	*“They [ACHIEVE scholars] took ownership and ran with it. We had two meetings after our initial meeting just to check in. The initial one was to check in about the prototype and after that gave them free rein to do work. I will say that it was clear that the level of research capacity was very high. I didn’t feel the need to double check. Even something like the recipes where there was a lot of thoughtfulness put into it.”* *“We had two deliverables: one infographic was something that we had as a deliverable for close to a year. It was done beautifully. The other piece – report on different funding streams – had just come up. The ACHIEVE opportunity came up.”*
Community impact of partnership	Addressed local issues	*“One of the biggest takeaways [from literature review on digital divide] is the number of young people that weren’t showing up to classes, weren’t showing up to programming, and being able to share that with youth center]…understand that there has to be some meeting our youth where they are. The nature of our work has to change.”*
	Increased community engagement	*“Their [ACHIEVE scholars’] flyers [for community garden], their social media – what they’ve created – is what I’m posting, and we’ve had – since I’ve been working with you all. I’ve had an increase of at least 30 new people on our message center, which is almost at a point of doubling from last year.”* *“I think this issue brief is going to help us to reach out to Black community/other communities of color to see if we can expand services.”*

### Community Outcomes

In 11 semi-structured interviews, community organizations that partnered with ACHIEVE scholars shared positive experiences from the partnership (Table 3). Some partners reported that the scholars provided diverse ideas and expertise. One organization leader described the scholars as “thought partners,” explaining: “*They asked a lot of questions that allowed us to think about how we want to approach the deliverables. That consistent communication was really helpful*.” Additionally, some partners mentioned that scholars reduced the workload for the organization. For example, one organization leader shared, *“If we hadn’t partnered with your team, I don’t think we would have gotten this issue brief done in such a timely manner.”* Lastly, organization leaders indicated that having the scholars produce specific deliverables of high quality was impactful as it enabled the organization to disseminate such materials to their own partners as well as community members.

In addition to ACHIEVE’s impact on the specific community partners, organization leaders also highlighted the overall community benefits from this partnership. They indicated that the deliverables from the project help address specific local issues. For example, scholars created infographics for a community organization to gather support for expanding and sustaining their early literacy program in Chicago pediatric clinics. One leader from the respective program shared, “*The ultimate goal [of the infographics] was to tell the story of the impact of our program to help inspire people to get more involved… There are over 110 sites…Those represent about 2000 medical providers as well as the families that they serve…Hopefully it tells the right story for funders…and would inspire them to give.”* Additionally, the partnerships allowed for increased engagement within communities. One leader shared, *“The issue brief on racial practices in lung measurement is going to help us reach out to more Black communities, other communities of color to see if we can expand services.”*

## Discussion

ACHIEVE was an innovative program that benefited both youth and community organizations. Through classroom-based learning and civic engagement opportunities, scholars were trained and empowered to make meaningful changes in their communities. The partnerships between scholars and community organizations were symbiotic. Scholars gained hands-on experiences in addressing local issues and produced practical deliverables relevant to current community issues, while community organizations learned from scholars and advanced their missions through the projects and resulting deliverables.

The ACHIEVE program resulted in positive developmental outcomes for scholars. The program’s design allowed scholars to learn about disparities through expert-led seminars as well as apply newly acquired knowledge and skills to community-based projects with peers and community organizations. Increasing awareness of inequities is a stepping stone toward building community capacity to effectively address community issues and ultimately improving inequities [16–18]. Along with helping enhance youth understanding of community issues, the ACHIEVE program provided the resources and environment for scholars to develop skills that are transferrable in both educational and professional pursuits, an important finding as youth, especially of marginalized populations, struggle to access such educational opportunities [19–21]. Lastly, the ACHIEVE program instilled a sense of empowerment and responsibility among scholars to engage with and support local communities. These experiences can be valuable in shaping their educational and professional aspirations, especially for high school scholars who are often in the first generation to go to college.

The outcomes seen among scholars in the ACHIEVE program align with other youth development programs based in Chicago that focus on health topics, including the CHAMPIONS NETWork and Youth Health Services Corps. The CHAMPIONS NETWork led to increased knowledge and self-efficacy among program participants who were trained to be health advocates within their communities [22]. For the Youth Health Services Corps, which engaged immigrant youth of Latino descent in community-based participatory research, an evaluation showed that participants had interest in continuing to deliver health education to community members [23]. Such programs have demonstrated benefits for youth and support their meaningful work within communities. Thus, greater investment in youth programs is needed, particularly in marginalized communities that have potential to benefit from more opportunities for youth and community development.

While the ACHIEVE program’s primary objectives focused on scholar outcomes, it also prioritized the development of meaningful partnerships with community organizations to effect local issues. According to the community organization leaders, ACHIEVE scholars were valuable assets who advanced organizational missions and produced deliverables that positively impacted communities. While non-profit organizations may struggle to build capacity, the ACHIEVE program demonstrated that engaging youth and establishing training to facilitate partnerships and projects with community organizations can be a viable strategy to support local organizations [24]. To ensure the success of partnerships in the ACHIEVE program, it was imperative to create an infrastructure for the work, for example by sharing project management tools with scholars, developing project proposals with organization leaders to ensure alignment among the organization and program as well as their timelines, and providing platforms for regular communication between the organization contact and scholars. Although local programs like the Youth Health Services Corps partnered with community organizations throughout their study, they did not capture the organizational impact from youth engagement, as described here [23]. 

Although the ACHIEVE program demonstrated positive outcomes, the program had limitations related to participant engagement. The varied engagement of high school scholars impacted our evaluation, as noted by the low survey response rate among high school scholars (22.5%). As such, it is important to note that the findings may not fully capture the impact of the ACHIEVE program on high school scholars and opportunities to refine the program to accommodate high school scholars. Further, the high school scholars selected for the ACHIEVE program likely have had the confidence, resources, and support to apply for the program, thus the impact of the program may not be generalizable to all high school students. This program was also conducted in an urban setting within communities that have majority Black populations, which may affect generalizability of the findings. Finally, scholars and organizational leaders may have responded positively due to social desirability or perceived benefits. We attempted to minimize it by keeping surveys anonymous and providing clear directions in the surveys and interviews that we were looking to improve the program.

## Conclusion

The ACHIEVE program was successful in training and empowering young people to grapple with local health disparities through classroom-based learning and civic engagement. The program enhanced scholars’ knowledge, skills, and self-efficacy to impact their communities and community organization’s capacity to address the needs of their communities. Future work should assess the long-term impacts of the program on scholars and community organizations. The findings suggest that this program has a positive impact, can be easily adapted for successful implementation within vulnerable communities, and can be tailored to address local community needs.

## Data Availability

The data that support the findings of this study are available from the corresponding author upon reasonable request.
